# How to resolve cryptic species of polypores: an example in *Fomes*

**DOI:** 10.1186/s43008-019-0016-4

**Published:** 2019-09-23

**Authors:** Ursula Peintner, Regina Kuhnert-Finkernagel, Viana Wille, Franco Biasioli, Anton Shiryaev, Claudia Perini

**Affiliations:** 1 University Innsbruck, Institute of Microbiology, Technikerstr. 25, 6020 Innsbruck, Austria; 2 Food Quality and Nutrition Department, Edmund Mach Foundation, Via Edmund Mach 1, 38010 San Michele all’ Adige, Italy; 3 Vegetation & Mycobiota Diversity Department, Institute of Plant and Animal Ecology (IPAE), Ural Branch of the Russian Academy of Sciences (UrB RAS), 8 March str., 202/3, 620144 Ekaterinburg, Russia; 4 Department of Life Sciences, University Siena, 53100 Siena, Italy

**Keywords:** Wood-degrading polypores, Volatile organic compounds, Mycelial growth rates, Chemotaxonomy, Morphological character evaluation

## Abstract

Species that cannot be easily distinguished based on morphology, but which form distinct phylogenetic lineages based on molecular markers, are often referred to as cryptic species. They have been proposed in a number of fungal genera, including the basidiomycete genus *Fomes*. The main aim of this work was to test new methods for species delimitation in cryptic lineages of polypores, and to define useful characters for species identification.

A detailed examination of a number of different *Fomes* strains that had been collected and isolated from different habitats in Italy and Austria confirmed the presence of distinct lineages in the *Fomes fomentarius* clade. Our zero hypothesis was that the Mediterranean strains growing on *Quercus* represent a species which can be delimited based on morphological and physiological characters when they are evaluated in statistically relevant numbers*.* This hypothesis was tested based on phylogenetic analysis of the rDNA ITS region, morphological characters of basidiomes and pure cultures, growth rates and optimum growth temperature experiments, mycelial confrontation tests, enzyme activity tests and volatile organic compound (VOC) production. The Mediterranean lineage can unambiguously be delimited from *F. fomentarius*. A syntype of an obscure and previously synonymized name, *Polyporus inzengae*, represents the Mediterranean lineage that we recognize as *Fomes inzengae*, a distinct species. The rDNA ITS region is useful for delimitation of *Fomes* species. Moreover, also a variety of morphological characters including hymenophore pore size, basidiospore size, and diameter of skeletal hyphae are useful delimiting characters. The ecology is also very important, because the plant host appears to be a central factor driving speciation. Physiological characters turned also out to be species-specific, e.g. daily mycelial growth rates or the temperature range of pure cultures. The production of VOCs can be considered as a very promising tool for fast and reliable species delimitation in the future.

## INTRODUCTION

*Fomes fomentarius* sensu *lato* (*s. lat.)* is thought to be a polypore taxon with a wide distribution in Europe, Asia, Africa, and North America. It is commonly known as the “tinder fungus”, “hoof fungus”, “tinder conk”, “tinder polypore”, or “Iceman’s fungus”. The 5000-year-old Iceman probably used this polypore: to make and preserve fire, as a first aid kit, an insect repellent, or for spiritual purposes (Peintner et al. 1998; Pöder & Peintner 1999). Besides the widespread and important use as tinder, *F. fomentarius* was a valued medicinal polypore in European traditional medicine. Its use as a styptic persisted throughout medieval times and it was prescribed as a remedy against dysmenorrhoea, haemorrhoids, and bladder disorders; the active substance being “fomitin” (Killermann 1938). Grienke et al. (2014) extensively reviewed the applications of *F. fomentarius* in traditional medicine and the current knowledge on its metabolite profile. Recent phylogenetic analyses based on multiple genetic markers indicated that *F. fomentarius* possibly contained cryptic species (Pristas et al. 2013). Our earlier study also indicated that a European lineage could possibly represent a separate species that could be differentiated based on growth characteristics and substrate differences (Dresch et al. 2015). The main aim of this work is to thoroughly investigate multiple vouchers and strains of the *Fomes fomentarius s. lat.* lineage in order to find meaningful and representative characters for the reliable distinction and differentiation of species representing different lineages. Molecular phylogenetic analysis, tests on growth characteristics, enzyme assays, and comparative analysis of volatile compounds, were carried out for this purpose. Moreover, we set high values on morphological characteristics of the basidiomes and of mycelia because they are crucial characters for an easy, fast and correct identification of fungal basidiomes. Our results clarify which methods and characters are most useful for distinguishing otherwise “cryptic” species in polypores.

## MATERIALS AND METHODS

### Sampling sites and environmental data

*Fomes fomentarius s. lat.* was sampled in different habitats in Austria (Tyrol) and Italy (Tuscany). Voucher numbers, plant hosts, as well as habitat are given in Table 1.

**Table 1:** *Fomes* sequences included in the phylogenetic analysis with information on the species identification, the newly sequenced voucher, the GenBank Accession number, and available information on geographic provenance as well as on host plant and isolation source. Sorted based on GenBank Accession number within clades

*Fomes species*	Voucher	GenBank	Country	Host	Isolation source
*F. inzengae*	*Erb. critt. Ital.* no 636, SIENA, (within Mycotheca Universalis) lectotype		Italy, Sicilia, Palermo	*Populus nigra*	basidiome
*F. inzengae*	*Erb. critt. Ital.* no 977, SIENA, (within Mycotheca Universalis)		Italy, Campobasso, San Giuliano dal Sanno	*Quercus*	basidiome
*F. inzengae*		AM981233	Slovenia Notranjska	*Abies alba*	discoloured wood, silver fir
*F. inzengae*		AY849305	Italy	*Platanus x acerifolia*	basidiome
*F. inzengae*		AY849306	Italy	*Platanus x acerifolia*	basidiome
*F. inzengae*		FJ865439	Slovakia	*Populus sp.*	basidiome
*F. inzengae*		FN539043	U.K. Wales	Angiosperm trees	wood
*F. inzengae*		FN539045	U.K. Wales	Angiosperm trees	wood
*F. inzengae*		GQ184602	Slovakia	*Fagus sylvatica*	basidiome
*F. inzengae*		GQ184604	Slovakia	*Populus alba*	basidiome
*F. inzengae*		GU731551	unspecified	*?*	?
*F. inzengae*		HM136673	France Grenoble	*Festuca paniculata*	plant roots
*F. inzengae*		HQ189535	Slovakia	*Cerasium avum*	basidiome
*F. inzengae*		HQ189535	Slovakia	*Cerasus avium*	basidiome
*F. inzengae*		JF927882	Italy	*Oreorchis patens (?)*	discoloured wood
*F. inzengae*		JX910366	China Kashgar Xinjiang Uyghur Autonomous Region	?	basidiome
*F. inzengae*	IB20130033	KM360129	Italy Siena Radicondoli Riserva Naturale Cornocchia	*Quercus cerris*	basidiome
*F. inzengae*		KM433840	Iran	Salix	basidiome
*F. inzengae*		KX426954	Poland Demanovsk	?	Demanovska Ice Cave air
*F. inzengae*		KX578020	Russia	?	basidiome
*F. inzengae*		LT629714	Italy Pisa Giardino Scotto	*Platanus*	basidiome
*F. inzengae*		MG719674	Switzerland	*Aesculus hippocastanea*	tree sucker
*F. inzengae*		MG719676	Switzerland	*Aesculus hippocastanea*	tree sucker
*F. inzengae*		MG719678	Switzerland	*Aesculus hippocastanea*	tree sucker
*F. inzengae*	IB20160349	MK184456	Italy Siena Monticiano Riserva Naturale di Tocchi	*Castanea sativa*	basidiome
*F. inzengae*	IB20160351	MK184457	Italy Siena Monticiano Riserva Naturale di Tocchi	*Carpinus betulus*	basidiome
*F. inzengae*	IB20160343	MK184458	Italy Siena Radicondoli Riserva Naturale Cornocchia	*Quercus cerris*	basidiome
*F. inzengae*	IB20160350	UDB034500	Italy Siena Monticiano Riserva Naturale di Tocchi	dead deciduous tree*, Castanea sativa, Quercus cerris*	basidiome
*F. inzengae*	IB20160342 epitype	UDB034501	Italy Siena Radicondoli Riserva Naturale Cornocchia	*Quercus cerris*	basidiome
*F. fasciatus*		JX126900	U.S.A. Louisiana	*Platanus occidentalis*	basidiome
*F. fasciatus*		JX126901	U.S.A. Georgia	*Quercus sp.*	basidiome
*F. fasciatus*		JX126906	U.S.A. Mississippi		basidiome
*F. fasciatus*		JX126907	U.S.A. Mississippi		basidiome
*F. fasciatus*		JX126908	U.S.A. Mississippi		basidiome
*F. fomentarius*		EF155492	Germany	*Fagus sylvatica*	wood
*F. fomentarius*		EF155493	Germany	*Fagus sylvatica*	wood
*F. fomentarius*		EF155495	Germany	*Fagus sylvatica*	wood
*F. fomentarius*		EU162056	Germany		wood
*F. fomentarius*		FJ865440	Slovakia	*Acer negundo*	basidiome
*F. fomentarius*		GQ184603	Slovakia	*Fagus sylvatica*	basidiome
*F. fomentarius*		GU062198	Latvia	*Alnus incana*	decayed wood
*F. fomentarius*		GU203514	unspecified		basidiome
*F. fomentarius*		HQ189534	Slovakia	*Fagus sylvatica*	basidiome
*F. fomentarius*		JF927720	Poland	*?*	?
*F. fomentarius*		JQ901965	Russia Moscow region	*Populus sp.*	?
*F. fomentarius*		JQ901966	Russia Moscow region	*Betula sp.*	?
*F. fomentarius*	IB20130011	KM360125	Austria Tyrol Innsbruck	*Picea abies stump*	basidiome
*F. fomentarius*	IB20130016	KM360126	Austria Tyrol Innsbruck	*Picea abies stump*	basidiome
*F. fomentarius*	IB20130019 Epitype	KM360127	Austria Tyrol Zirl	*Fagus sylvatica*	basidiome
*F. fomentarius*	IB20130022	KM360128	Austria Tyrol Innsbruck	*Picea abies stump*	basidiome
*F. fomentarius*		KM396269	Austria Tyrol	*Betula sp.*	basidiome
*F. fomentarius*	IB20170012	MK184459	Austria Tyrol Achenkirch	*Fagus sylvatica*	basidiome
*F. fomentarius*	IB20140121	MK295658	Italy, Campania, Parco del Cilento	*Fagus sylvatica*	basidiome
*F. sp. Asia*		DQ513402	China		?
*F. sp. Asia*		DQ513402	China Changbai Shan		basidiome
*F. sp. Asia*		EU273503	unspecified		?
*F. sp. Asia*		JX290073	unspecified		basidiome
*F. sp. Asia*		KJ668550	South Korea Odaesan National Park		?
*F. sp. Asia*		MH114657	China		?
*F. sp._Iran*		MK050587	Iran Darab kola		?
F. fomentarius II		JX126886	U.S.A. Alaska	*Betula neoalaskana*	basidiome
F. fomentarius II		JX183719	U.S.A. Minnesota	*Betula sp.*	basidiome
F. fomentarius II		JX183720	U.S.A. North Carolina	*Betula alleghaniensis*	basidiome
F. fomentarius II		HM584810	unspecified		?
F. fomentarius II		KC505546	unspecified		?

Sampling sites, basidiome morphology, and ecology (substrate) were documented in situ before collecting the basidiomes. Colours were documented based on the colour code of Cailleux (1986). Basidiomes were wrapped in greaseproof paper and transported to the laboratory for isolation. Basidiomes where then dried at 40 °C on a mushroom dryer, and vouchers deposited in the mycological collection in IBF.

### Isolation

Sterile techniques were used to obtain cultures from the context tissue of the basidiomes. Small pieces (2.0 mm^3^) were excised from each basidiome, plated on 2–3% w/v malt extract (MEA) agar plates and incubated for 1 to 3 weeks at 20 °C. Cultures were checked regularly for contaminants. Mycelial plugs 1–3 mm diam were taken from the edge of the mycelium and transferred to new plates to establish pure cultures and carry out growth experiments.

The tissue cultures and stock cultures are maintained at the Institute of Microbiology, University of Innsbruck, Austria. For cryopreservation, small parts of well-growing cultures were overlaid with 10% skimmed milk and stored at − 80 °C. Isolates were also stored on MEA slants at 4 °C.

### DNA amplification and sequence analysis

Molecular identification of the fungal isolates was performed using the barcoding ITS regions of the ribosomal DNA. DNA amplification was carried out from *Fomes* pure culture isolates. A direct colony PCR was performed on pure cultures that were about 1 week old as previously described (Walch et al. 2016). Alternatively, total genomic DNA was isolated from 100 μg of fungal matter (one-month-old mycelial cultures) by DNeasy® Plant Mini Kit (QIAGEN, Germany) according to the manufacturer’s instructions and then eluted in 50 μl of sterile water. ITS-1, 5.8S rDNA and ITS-2 regions were amplified in a 50 μl volume reaction containing 1–10 ng of genomic DNA, using the primers pair ITS1 / ITS4, and the LSU was amplified with the primers NL1 / NL4 in a T gradient Thermal Cycler (primus 96; Peqlab, Germany) according to Peintner et al. (2001). PCR products were sequenced by Microsynth AG (Switzerland) with all primers. Sequences were analysed using the Sequencher® software (version 5.2.3; Gene Codes, Ann Arbor, MI, USA).

As a first step, BLAST searches were conducted in GenBank (http://ncbi.nlm.nih.gov), and closely related sequences downloaded. Only a small part of identical sequences were downloaded in order to cover geographical range and substrate preferences.

Alignment and phylogenetic analyses were carried out with MEGA 6.0 (Tamura et al. 2011). The best Maximum Likelihood (ML) model was tested before carrying out a ML analysis. The analysis involved 60 nucleotide sequences. All positions with less than 90% site coverage were eliminated. There were 515 positions in the final dataset. *Fomes fasciatus* was used as outgroup. To evaluate branch robustness of trees, parsimony-based bootstrap analyses were applied. Bootstrap analyses were conducted Subtree-Pruning-Regrafting (SPR) algorithm level 5 in which the initial trees were obtained by the random addition of sequences (five replicates). For the BP search, all positions with less than 100% site coverage were eliminated.

Bayesian Inference in MrBayes 3.2.6 (Huelsenbeck and Ronquist 2001, Ronquist et al. 2012) was also used to test branch robustness. For prior probability settings, defaults were kept. For the Markov Chain Monte Carlo (MCMC) analyses, four chains were run for 10 million generations, with trees being sampled every 5000 generations. The analysis was stopped as the convergence diagnostic (average standard deviation of split frequencies) was below 0.05 after 10 million generations. From the 20,000 sampled trees (for each of the two runs) 25% were discarded as burn-in before summary statistics were calculated (using sump and sumt commands). Diagnostic plots, as well as the convergence diagnostics EES (Estimated Sample Size; min ESS around 10 K) and PSRF (Potential Scale Reduction Factor; 1000 for all parameters), indicated stationarity. Trees were drawn using FigTree 1.4.3. The newly created sequences were submitted to GenBank (Table 1).

### Microscopical analysis

Vouchers and pure culture isolates (2% MEA) were examined by means of standard microscopic techniques in 3% KOH, water, Melzer’s reagent, Congo red, and Cotton blue. Microscopic documentation and measurements were made with a Nikon NS Fi1 camera and the computer program NIS Elements 4.13. All measurements were made at 1000 fold magnification. At least 30 spores or hyphal elements were measured for statistical evaluation.

### Colony growth temperature experiments

All strains were first cultivated on plates containing 25 mL Malt Extract Agar (3% MEA), in order to ensure the same starting conditions for all strains. After 7 d, four mycelia plugs (5 mm diam.) were taken 1 cm from the leading edge of the colony and transferred to the middle of plates of 9 cm diam containing 25 mL MEA. Plates were randomly placed into a plastic box, and incubated at seven different temperatures (10, 20, 25, 30, 32, 35, and 37 °C). Mean colony diameter (mm), minus the 5 mm plug, was measured after 2, 5, 7 and 10 d. The results are expressed as means ± standard deviations of three parallel cultures.

### Drop test for enzymatic activity

Drop tests were used to test for important enzymes of wood decaying fungi, especially for laccases, polyphenol oxidases, and peroxidases. Drop tests were carried out as described in Taylor (1974) with modifications (Gramss et al. 1998). Test solutions were prepared as described by Gramss et al. (1998). Briefly, for the laccase test, 0.1 M α-naphthol was dissolved in 96% denatured ethanol; with positive laccase reaction, the colour of the fungal tissue changes into blue or violet. For the phenol oxidase test, 2.5% gum guaiac was also dissolved in 96% denatured ethanol. When phenol oxidases like catechol oxidase, laccase and monophenol monooxygenase are present, the colour changes to very dark green. The peroxidase test was carried out as pyrogallol(+) or pyrogallol(−) test: for the pyrogallol(−) test, 0.5% pyrogallol diluted in water (w/w) was applied; for the pyrogallol(+) test, pyrogallol was supplemented with a drop of the 0.2% H_2_O_2_. Both pyrogallol tests formed a brownish colour, when reacting with peroxidases. For the drop test, petri dishes containing one pure culture isolate growing for 10 d at 20 °C were used. Petri dishes were divided into four sections, each treated with one test. The colour reactions and their intensities were observed and documented after 1, 3 h for α-naphthol and gum guaiac, and after 24 h for pyrogallol.

### Mycelial confrontation tests

Mycelial confrontation tests were performed based on the heterokaryotic hyphae isolated from *Fomes* basidiomes. Two mycelial plugs were placed opposite to each other on an agar dishes containing 2% MEA. All possible combinations of the two *F. fomentarius* (IB20130019, IB2013022) and the Mediterranean (subsequently identified as *F. inzengae*) strains (IB20160349, IB20160351) were tested. Petri dishes were incubated at 25 °C for 6 d. Results of their compatibility were then documented photographically and evaluated in four qualitative categories: very weak, weak, medium, strong interaction.

### Analysis of volatile metabolites

Volatile compounds analysis was performed by a Proton Transfer Reaction Time of Flight Mass Spectrometer (PTR-TOF-MS; PTR-TOF 8000, Ionicon Analytik, Innsbruck, Austria) according to the procedure described in Khomenko et al. (2017). Ensuing spectra were treated and analysed according to Cappellin et al. (2012).

One part of the samples was taken from the air-dried basidiome context in the area of the youngest pore layers. Samples were finely ground by an IKA mill under liquid nitrogen. From the resulting powder, 0.1 g was mixed with 3 mL milli Q water in closed glass vials and left for 6 h at 8 °C. The samples were then incubated at 40 °C for 30 min. and measured for 1 min.

Analysis was also performed on freeze-dried mycelial pure cultures grown for 3 wk. on MEA 3% at 25 °C. Depending on the amount of harvested mycelium, between 7 and 11 mg were used for the analysis. The mycelium was soaked in 1 mL milli Q water in closed glass vials for 6 h at 8 °C. The samples were then incubated at 40 °C for 30 min. and measured for 1 min. This second analysis was carried out to test for a potential influence of the different types of wood substrates of the basidiomes.

### Statistics

Data analysis was carried out with Statistica 9.1 (StatSoft 2010) for Windows 10. Data are given as arithmetic means with standard deviations. Variables were tested for normal distribution. Parameters with normal distribution were compared by t-tests (or Mann-Whitney U Test if data show no variance homogeneity). Differences in colony growth development after 5 d by different incubation temperatures were tested using the one-way ANOVA and Tukey HSD test. If parameters were not normally distributed, the one-way ANOVA was replaced by the Kruskal-Wallis one-way analysis of variance on ranks. Significance value for all tests was *p* < 0.05. Unsupervised PCA (Principal Component Analysis) and Kruskal-Wallis one-way analysis of variance on ranks of PTR-TOF-MS data were performed by R (R Core Team 2017).

## RESULTS

### Phylogenetic analysis

**Fig. 1:**
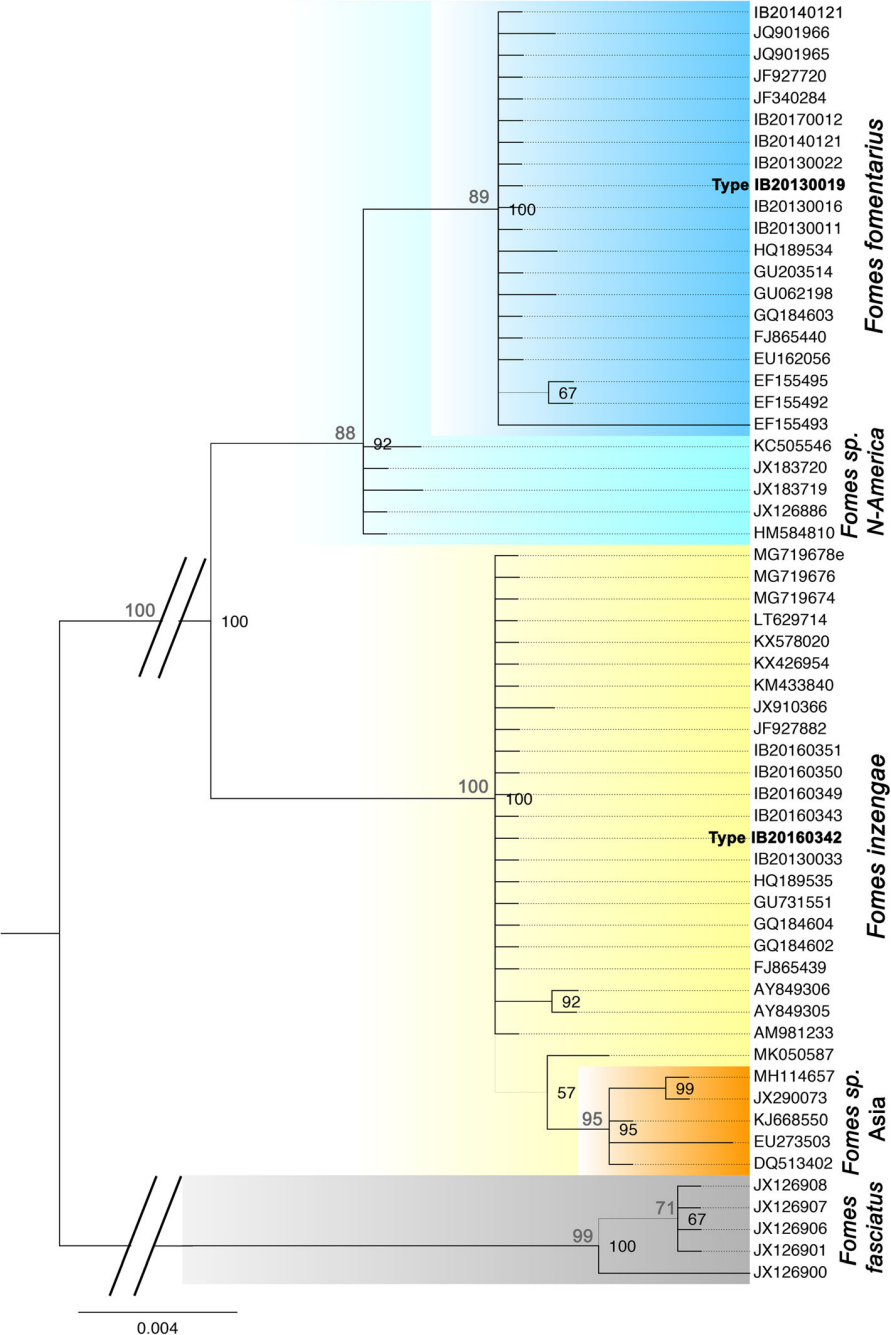
ITS-based Bayesian phylogeny of *Fomes fomentarius s. lat* rooted with *F. fasciatus*. Maximum Parsimony bootstrap values > 70% appear above the branches in grey. Bayesian probabilities > 65% appear in black, right of the respective node. Grey branches in the phylogeny are not supported. *Fomes inzengae* is strongly supported as a distinct species

Phylogenetic analyses were performed with 60 rDNA ITS sequences obtained from our *Fomes* isolates and selected sequences currently available in public databases (GenBank). After a test for the best ML model, a Hasegawa-Kishino-Yano model was used for the ML analysis. The ML tree with the highest log likelihood (− 1143.4536) is in accordance with the Bayesian tree (Fig. 1). Bootstrap values were calculated with Maximum Parsimony (500 replicates), and the four most parsimonious trees (length = 83) were obtained with a Consistency Index of 0.951613, a Retention Index of 0.993890, and a Composite Index of 0.955663 for parsimony-informative sites.

The phylogenetic tree allows for the distinction of two well-supported major lineages within the *F. fomentarius* species complex in Europe, representing *Fomes fomentarius* and another *species* of *Fomes*. The four strains isolated from the alpine range fall within a clade of *F. fomentarius* sequences originating in Northern European countries (Russia, Poland, Latvia, Slovak Republic, Germany, Austria, Slovenia). Also, a strain from southern Italy growing on *Fagus* falls in this clade (IB20140121). Typical plant substrates are *Fagus sylvatica, Alnus* spp., *Acer negundo,* and *Picea abies*. We consider this lineage as the *Fomes fomentarius s. str.* Lineage. It is sister to a clade from North America growing on *Betula* spp., probably representing another species of *Fomes*.

The sequences from the other European *Fomes* isolates cluster within a clade of *Fomes* sequences originating mostly from central to southern European countries (Italy, France, Portugal, Slovenia). In this case the plant substrates are *Aesculus, Carpinus, Cerasium*, *Platanus, Populus* spp*., Quercus* spp*.,* and *Abies*. This clade has a close relationship to a clade of *Fomes* from Asia that might represent a fourth distinct species.

Internal clade sequence divergence was small, with 0–3 base pair differences between the different strains of *F. fomentarius s. str*. (0.02%), and 0–1 f base pairs between the Mediterranean (*F. inzengae)* sequences (0.01%) (ITS1–5.8S-ITS2 region). Sequence divergence between the *F. fomentarius s. str*. and the *F. inzengae* clade was 9–18 base pairs (2.6%). Sequence divergence of the latter both to the outgroup *F. fasciatus* was 41–62 base pairs. Thus, pairwise distances confirm that *F. fomentarius s. str*. and *F. inzengae* can be considered as two distinct sister taxa.

Phylogenetic analyses indicate a strong influence of the plant host substrate on speciation events in this genus of lignicolous, and opportunistically pathogenic basidiomycetes.

### Pore diameter

**Fig. 2:**
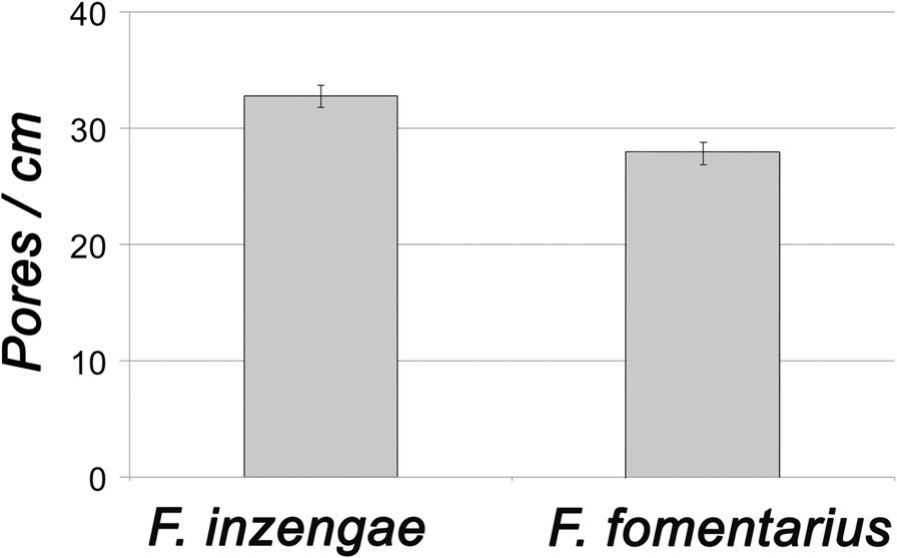
Comparison of pore diameter (as pores / cm hymenophore surface) of *Fomes inzengae* and *F. fomentarius*. Pore diameter is significantly different (*p* = 0.000027, *n* = 9)

The basidiomes of *F. fomentarius* have 27–30 pores / cm (MW ± SD: 27.9 ± 0.9 pores / cm, *n* = 9), those of recently collected *F. inzengae* have 31–34 pores / cm (MW ± SD: 32.8 ± 0.9 pores / cm, *n* = 9). Thus, the *F. inzengae* strains produced significantly smaller pores than *F. fomentarius* (*p* = 0.000027, *n* = 9) (Fig. 2). The mean pore diameter of *F. inzengae* was 0.31 mm, and of *F. fomentarius* 0.36 mm.

### Basidiospore size

Basidiospores of *F. inzengae* are 9–12.5 × 3–4 μm (mean length = 10.8 ± SD = 0.9, mean width = 3.3 ± SD = 0.3, mean Q = 3.3 ± SD = 0.3, *n* = 37). This is smaller than the basidiospore size of 12–18 (− 20) × 4.0–7.0 μm as reported for *F. fomentarius* (Ryvarden & Gilbertson 1993, 1994), or as measured from our materials.

### Mycelial characteristics in pure culture

Pure cultures of two strains, *F. fomentarius* IB20130016 and *F. inzengae* IB20160342, were comparatively investigated microscopically at all incubation temperatures. The best results were achieved with Congo red staining.

**Fig. 3:**
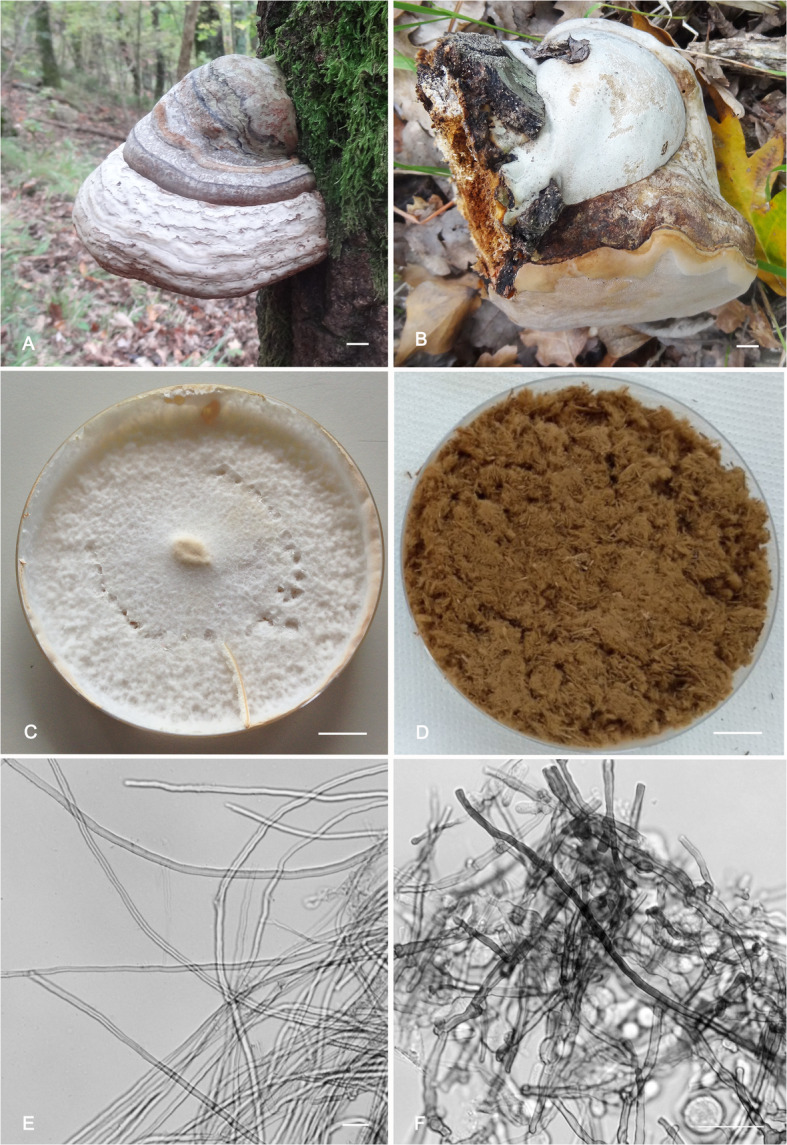
*Fomes inzengae*. A. Basidiome of the lectotype (IB20160342) growing on *Quercus cerris* in the Natural Reserve of Cornocchia. B. Basidiome with new hymenophore formation (positive geotropical reaction) after falling of the host tree (IB20160343). C. Mycelia pure culture after 10 d on 3% MEA at 25 °C (IB20160342). D. Ground basidiome (IB20160342); note the ferruginous brown colour and fluffy consistence. E. Skeletal hyphae as formed after 5 d  on 3% MEA at 37 °C (IB20160342). F. Inflated intercalar and terminal hyphal elements after 5 d at 37 °C, stained with Congo red (IB20160342). Bars A-D = 1 cm; E-F = 10 μm

**Fig. 4:**
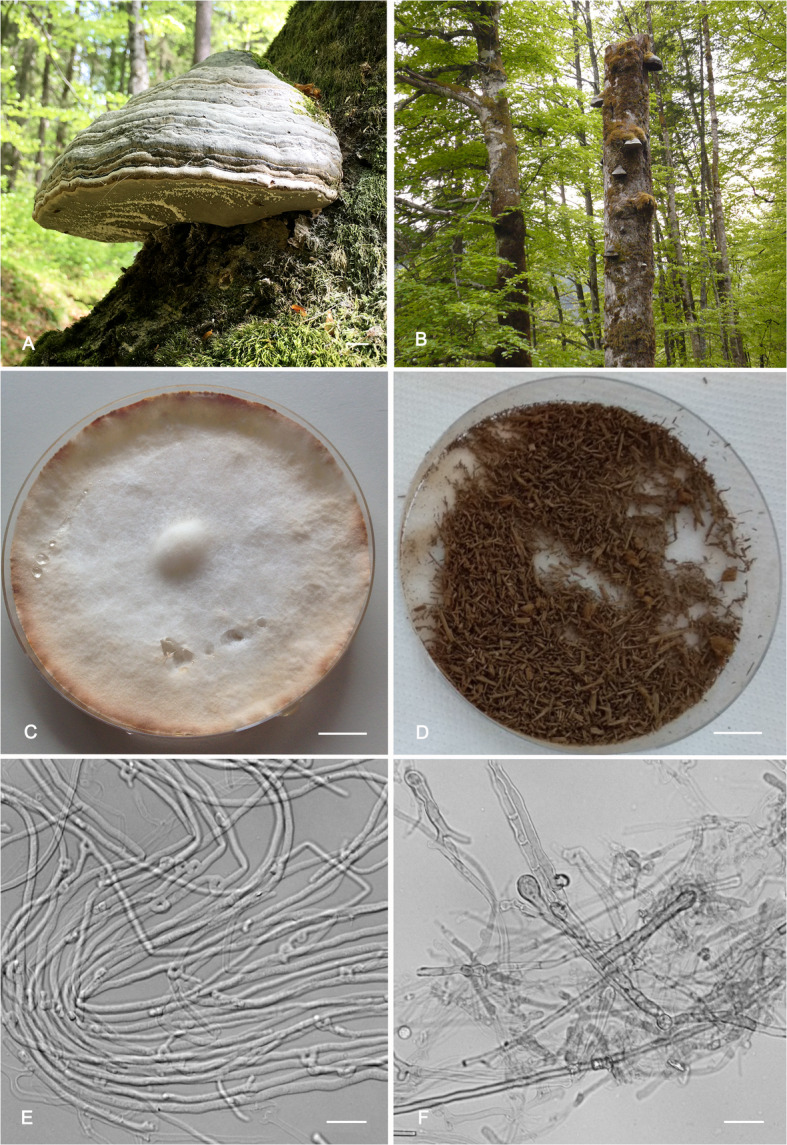
*Fomes fomentarius*. A. Basidiome growing on *Fagus sylvatica* in Tyrol (Austria) (IB20170012). B. Several basidiomes growing on a dead stem of *Fagus sylvatica*. C. Mycelia pure culture (IB20130016) after 10 d on 3% MEA at 25 °C. D. Ground basidiome (IB20170012); note the dark brown colour and granular consistence. E. Generative hyphae with clamp connections (IB20130016) as formed after 5 d on 3% MEA at 30 °C. F. Inflated intercalary and terminal hyphal elements (IB20130016) after 5 d at 37 °C. Bars A, C-D = 1 cm; E-F = 10 μm

A typical trimitic hyphal system was constantly established at all temperatures by both strains: skeletal hyphae, binding hyphae, and generative hyphae with clamp connections, were always present, only varying in the composition of the three types of hyphae from strain to strain and at different temperatures. At 32 °C and above, both strains formed inflated roundish terminal and intercalary hyphal elements up to 10 μm diam. *Fomes inzengae* formed these elements in greater quantities and more readily, already starting at 30 °C (Figs. 3 and 4).

### Differential characteristics of ground basidiomes

The powders resulting from ground basidiomes of *F. fomentarius* and *F. inzengae* could usually be differentiated by their consistency and pigmentation: the powder from *F. fomentarius* basidiomes was dark brown, and arenaceous / granular, whereas that of *F. inzengae* basidiomes was ochraceous brown and fluffy. However, there were also exceptions, such as a *F. inzengae* basidiome that could not be unambiguously identified based on this character (Figs. 3 and 4).

The basidiome powders also exhibited different behaviours when mixed with water: the *F. fomentarius* powder floated, while that from *F. inzengae* swelled like a sponge.

### Diameter of skeletal hyphae in pure culture and in basidiomes

**Fig. 5:**
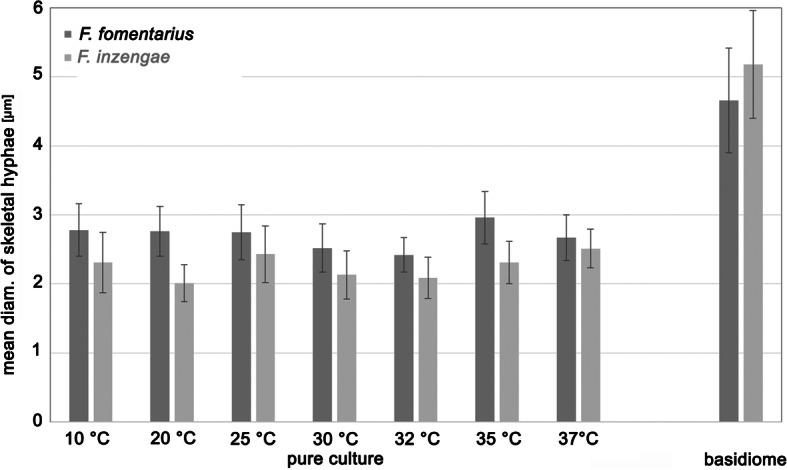
Diameter of skeletal hyphae in pure culture after 10 d incubation on 3% MEA at different temperatures and in naturally grown basidiomes. Differences between *F. fomentarius* and *F. inzengae* are always highly significant (p < 0.0001) with the exception of 37 °C (p < 0.05) (*n* = 45 for each temperature; *n* = 75 for *F. fomentarius* basidiomes; and *n* = 90 for *F. inzengae*)

The diameter of the skeletal hyphae was generally significantly different between *F. fomentarius* and *F. inzengae.* In pure culture, the skeletal hyphae of *F. fomentarius* ranged from 1.5–3.7 μm diam, and those of *F. inzengae* from 1.3–3.5 μm. Through all tested temperatures, *F. fomentarius* had broader skeletal hyphae than *F. inzengae*. This difference was highly significant for the incubation temperatures 10, 20, 30, and 35 °C (*p* = 0.000000, *n* = 45 for each temperature) The diameter of the skeletal hyphae appears to be temperature-dependent in pure culture (Fig. 5).

The skeletal hyphae of the basidiomes were always significantly wider than ones produced in pure cultures. In the basidiomes, the diameter of *F. fomentarius* skeletal hyphae ranged from 3.0–6.4 μm, and those of *F. inzengae* from 3.2–6.9 μm. Thus, *F. inzengae* produced significantly wider skeletal hyphae in the basidiomes than *F. fomentarius* (*p* = 0.000027, n_F.fom_ = 75, n_F.inz_ = 90) (Fig. 5). All *Fomes* strains developed thicker skeletal hyphae in the harvested basidiomes than in pure cultures. Interestingly, the differences between skeletal hyphae of the two species were always significant but reversed: in harvested basidiomes *F. inzengae* had wider skeletal hyphae than *F. fomentarius*, but in pure cultures *F. inzengae* had thinner ones than *F. fomentarius*.

### Colony growth at different temperatures

**Fig. 6:**
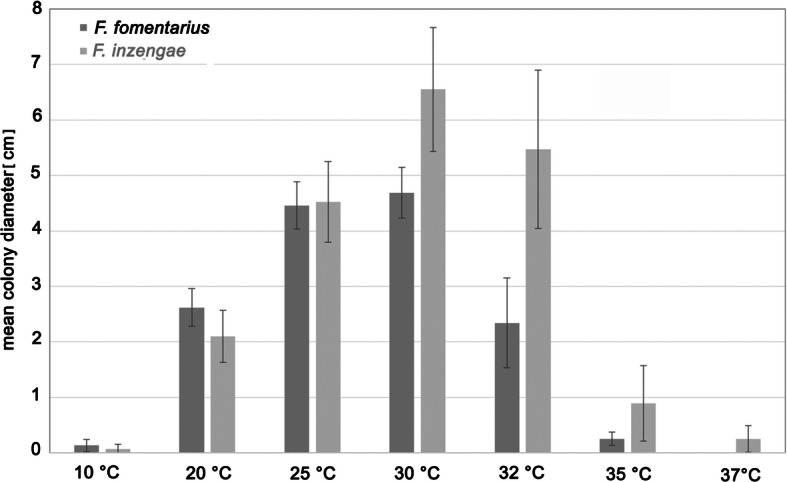
Mean colony diameter after 5 d on 3% MEA at different temperatures. *F. inzengae* grows significantly faster at temperatures of 30 °C and higher, but slower at 20 °C and below. With the exception of 25 °C, differences in growth rates between *F. fomentarius* and *F. inzengae* are always highly significant (*p* < 0.0001) (*n* = 45)

All *Fomes* strains grew well at temperatures of 25–30 °C, and did not show any significant difference at these temperatures. However, *F. inzengae* strains have a higher optimal temperature range of 30–32 °C. The performance of strains belonging to the two species at the other temperatures is clearly different: *F. fomentarius* strains grow significantly faster at 10 and 20 °C than the *F. inzengae* strains (10 °C: *p* = 0.018; 20 °C: *p* = 0.000010). At 25 °C, no significant difference could be detected, but a slight tendency of the *F. inzengae* strains to growing larger colonies was observed. At higher temperatures (30–37 °C), the *F. inzengae* strains grew significantly faster (30 °C: *p* = 0.000000; 32 °C: p = 0.000000; 35 °C: *p* = 0.000002; 37 °C; *p* = 0.000000) compared to *F. fomentarius* (Table 2, Fig. 6).

**Table 2:** Effects of temperature on mycelial growth (cm/day) of ten *Fomes* strains cultivated on 3% MEA. The mycelial growth rate per day [cm/day] was calculated for the first 7 days of incubation

*Fomes* species	voucher		Mycelial growth rate [cm/day]						
		10 °C	20 °C	25 °C	30 °C	32 °C	35 °C	37 °C	all T
*F. inzengae*	IB20130033	0.02	0.32	0.77	0.94	0.93	0.12	0.02	0.45
	IB20160342 Type	0.01	0.32	0.86	1.10	1.09	0.12	0.03	0.52
	IB20160349	0.02	0.39	0.83	1.01	0.98	0.35	0.09	0.52
	IB20160350	0.01	0.28	0.50	0.80	0.84	0.33	0.08	0.40
	IB20160351	0.03	0.49	0.87	1.01	0.81	0.12	0.03	0.48
	IB20160343	0.01	0.29	0.72	0.87	0.70	0.01	0.00	0.37
	Mean	0.02	0.35	0.76	0.95	0.89	0.19	0.04	0.46
	SD	0.01	0.08	0.14	0.11	0.14	0.13	0.04	0.06
*F. fomentarius*	IB20130011	0.01	0.41	0.70	0.83	0.67	0.05	0.00	0.38
	IB20130016	0.05	0.51	0.86	0.85	0.57	0.05	0.00	0.41
	IB20130019 Type	0.02	0.41	0.65	0.63	0.45	0.02	0.00	0.31
	IB20130022	0.03	0.46	0.79	0.79	0.51	0.06	0.00	0.38
	Mean	0.03	0.45	0.75	0.78	0.55	0.04	0.00	0.37
	SD	0.02	0.05	0.09	0.10	0.09	0.02	0.00	0.04

The mycelial growth rate per day was calculated for each isolate and the most relevant incubation temperatures (20, 25, 30, and 32 °C). This confirmed that *F. fomentarius* grows faster at 20 °C, and slower at 30 °C and 32 °C than *F. inzengae* strains. Strain properties appear to be important, as some strains (e.g. *F. inzengae* IB20160342) grow extraordinarily fast, and others extraordinarily slow (*F. fomentarius* IB20130019) (Table 2).

### Enzymatic activity

Laccase and phenol oxidase tests were always positive for all tested strains. Peroxidase tests gave ambiguous results and were dependent on the age of the pure culture rather than on the particular strain.

### Confrontation tests between heterokaryotic mycelia

**Fig. 7:**
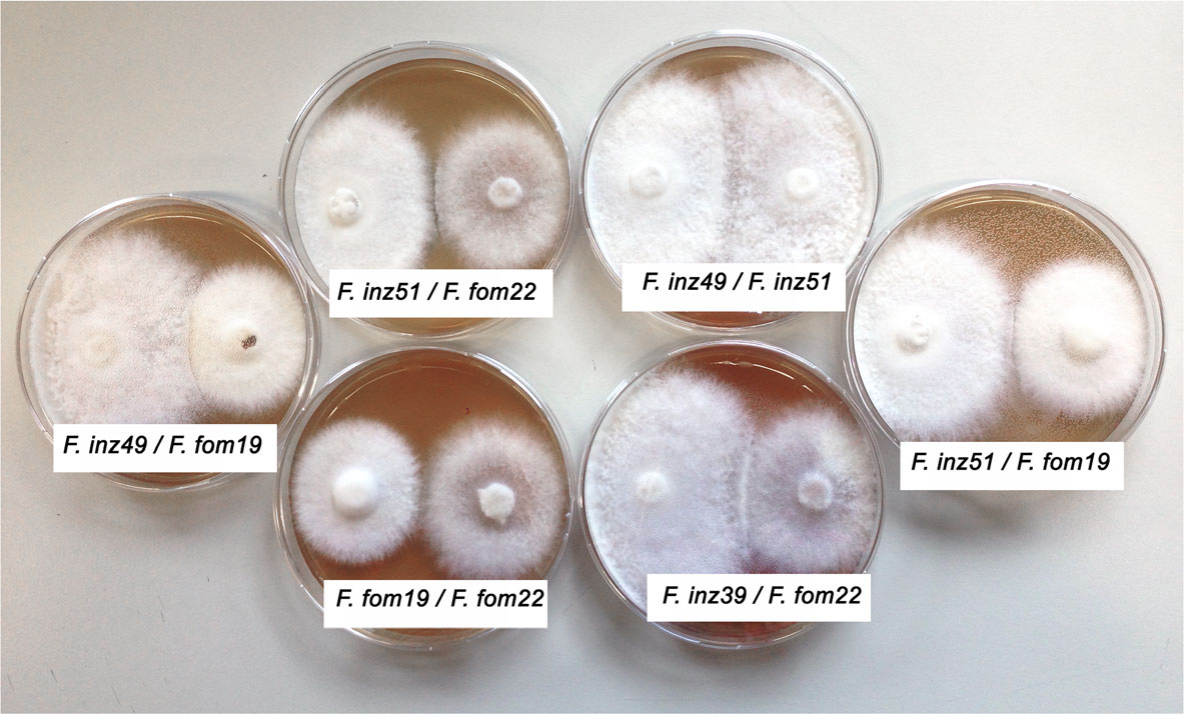
Confrontations test of different isolates of *Fomes fomentarius* and *F. inzengae* after 6 d on MEA 3% at 25 °*C. fomes inzengae* is always growing faster and with a fluffier surface. F. inz49 = *F. inzengae* (IB20160349), F. inz51 = *F. inzengae* (IB20160351), F. fom19 = *F. fomentarius* (IB20130019), F. fom22 = *F. fomentarius* (IB20130022)

These were carried out at 25 °C as at that temperature there are no significant differences in growth rates between the tested strains. When strains were tested against themselves, hyphal anastomoses were readily formed all over the confrontation zone (positive reactions). The strains tested (*F. fomentarius* IB20130019, IB20130022; *F. inzengae* IB20160349, IB20160351) did not show any kind of inhibition under the reflected-light microscope and grew easily into each other. However, when a strain was confronted with any other strain, the isolates formed distinct colony margins, and no anastomoses were formed. Overall, the *F. inzengae* strains were more competitive than *F. fomentarius* strains at 25 °C, and *F. fomentarius* strains always exhibited reduced growth whenever they were matched with any other strain (Fig. 7).

### Volatile metabolites

**Fig. 8:**
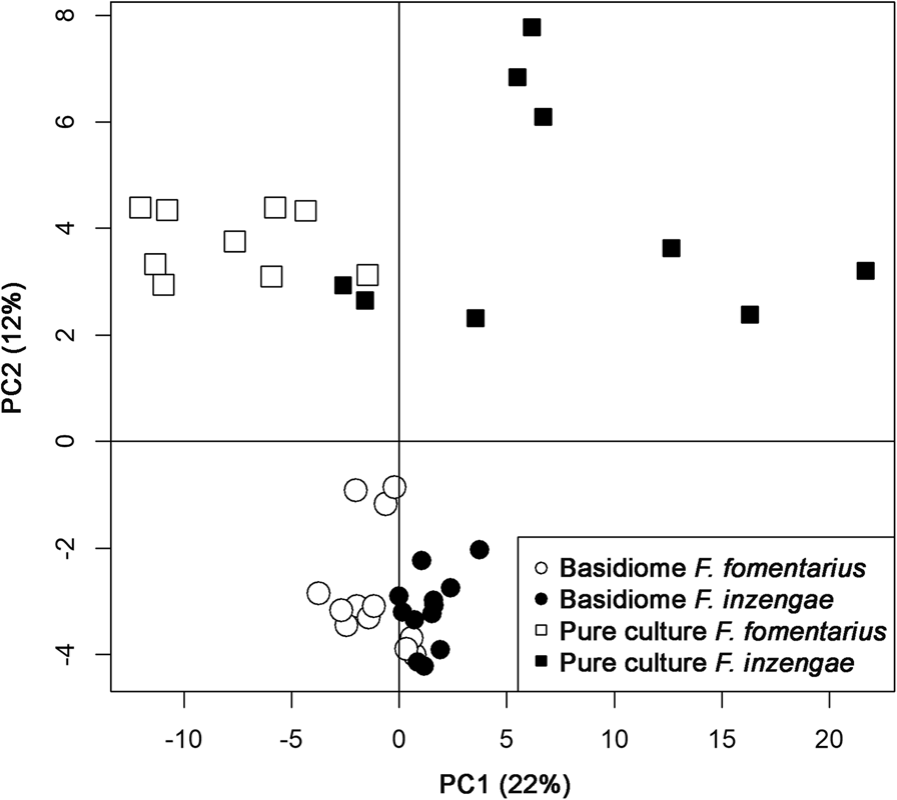
VOC data exploration by unsupervised PCA analysis of all *Fomes inzengae* and *F. fomentarius* samples (232 peaks). Basidiome and pure culture samples are well separated by the second principal component (PC2 12.5%). Separation of *F. inzengae* from *F. fomentarius* is more pronounced in pure culture samples than in basidiomes (PC1 22.9%)

**Fig. 9:**
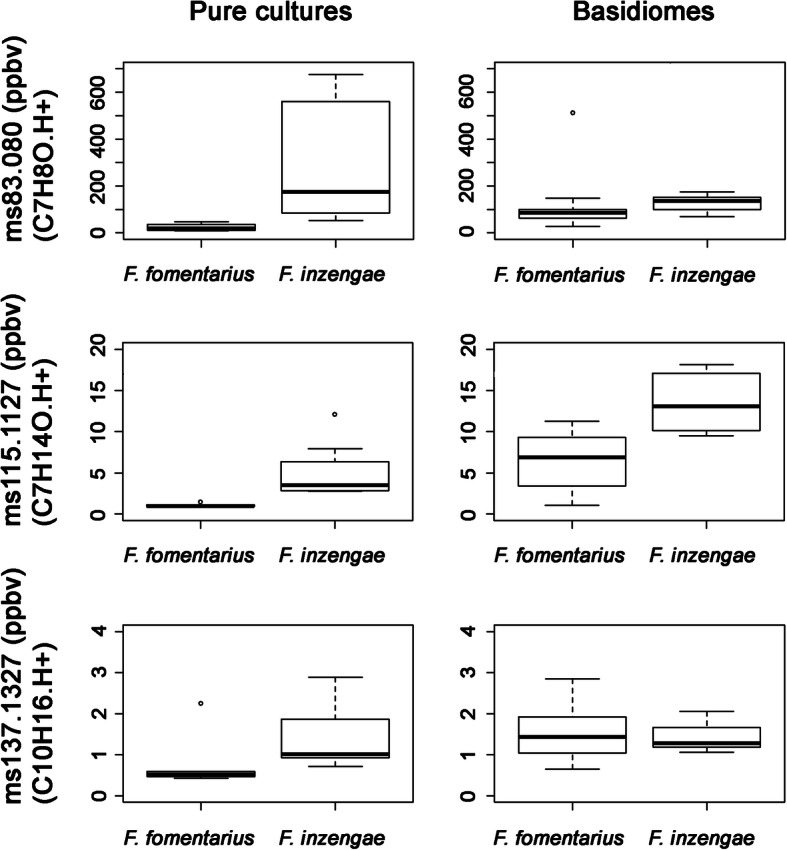
Three exemplar mass peaks with significantly different concentrations between *Fomes inzengae* and *F. fomentarius*: C4H8O.H+ (protonated butanal/butanone), C7H14O.H+ (protonated heptanal/heptanone) and C10H16.H+ (protonated monoterpenes) Pure culture samples had always better separation in VOCs concentration than basidiomes. The interaction with the substrate increases VOCs emission in *F. fomentarius*

The PTR-TOF-MS dataset contained more than 300 mass peaks. Peaks with a concentration significantly higher than blanks were 232 for basidiome samples and 209 for pure culture samples. Data exploration by unsupervised PCA analysis of all samples (232 peaks) is shown in Fig. 8. Different sample sets (basidiome and pure culture) are well separated by the second principal component. More interestingly, the first component indicates a certain separation of *F. fomentarius* from *F. inzengae* which is clearer for pure culture samples: despite the small amount of material used, freeze dried mycelial samples provided a better resolution and separation. Based on a Kruskal-Wallis one-way analysis of variance, 91 mass peaks were significantly different between the pure culture samples of *F. inzengae* and *F. fomentarius*. Again, despite the larger amount of material available for the analysis, only 19 mass peaks were significantly different for the basidiome samples. Figure 9 shows the concentration of a few selected compounds. *Fomes inzengae* is generally richer in VOCs than *F. fomentarius,* something true for many VOCs whose production is not dependent on the substrate such as some carbonyl compounds (Fig. 9, left and middle panels). However, as shown in data from naturally grown basidiomes, substrate or other environmental conditions result in differences in VOC production, as in the case of monoterpenes (Fig. 9, right panels). Thus, the two *Fomes* species are producing species-specific volatile metabolites but the interaction with the substrate can mask this differences.

## TAXONOMY

**Fomes inzengae** (Ces. & De Not.) Cooke, *Grevillea* **14** (69): 18 (1885).

*Basionym*: *Polyporus inzengae* Ces. & De Not., *Erb. critt. Ital.*, ser. 1: no. 636 [typeset description on label with specimen] (1861).

*Type*: **Italy**: *Sicilia*: Palermo, on *Populus dilatata,* 1860–1861, *Inzenga* [det. Cesati & De Notaris, *Erb. critt. Ital., ser.* 1 no. 636 [intermixed with “Mycotheca Universalis”] (SIENA – ***lectotypus hic designatus***; IF556590); *Prov. Siena*: Radicondoli, Riserva Naturale Cornocchia, on living *Quercus cerris*, 26 Oct. 2016, *U. Peintner & C. Perini* (IB20160342, ***epitypus hic designatus***; IF556625).

*Diagnosis*: Basidiomes macroscopically very similar to *F. fomentarius* from which it can be differentiated by the following characters: the pluriannual basidiomes have a hymenophore with 32–40 pores / cm; and the basidiospores are (9.0–) 10–12 (− 12.5) x (2.8–) 3.0–3.5 (− 3.8), Q = (2.8–) 3.0–3.6 (− 3.7) μm.

*Description*: *Basidiomes* perennial, sessile, ungulate, tough, woody, to 20 cm wide. Upper surface quickly developing a glabrous crust, grey (92LM) with a few dirty olivaceous spots (NP69), dull. Greyish coloured upper part the basidiome crust often conspicuously and irregularly marbled or brown-dotted. Marginal growth zone consisting of a distinctly zoned layer, zones 0.5–3 mm wide, in different shades of reddish brown (PR55), brown (NP67–69) or ochraceous brown (M70–71), minutely tomentose; transitional zone between ochraceous brownish zonate margin and grey older crust sometimes conspicuous and darker brown. Pore surface concave, pale brown, pores circular, 31–34 (− 38) pores / cm, with thick tomentose dissepiments. Tube layers indistinctly stratified, brown (PR59) and becoming stuffed; context tissue layer between the surface crust and the tubular layers, reddish brown (PR45), tough, azonate. Granular core developing at the upper part of the context, next to the substrate. *Basidiospores* cylindric**,** hyaline, smooth, not amyloid, (9.0) 10–12 (− 12.5) x (2.8–) 3.0–3.5 (− 3.8) μm, Q = (2.8-) 3.0–3.6 (− 3.7); *n* = 37; a large proportion germinate immediately. *Basidia* not observed. *Cystidia* not observed. *Hyphal system* trimitic, generative hyphae hyaline, thin-walled, with clamp connections, inconspicuous, 1.5–3.5 μm diam; Contextual skeletal hyphae thick-walled, non-septate, walls yellowish brown in KOH (3%), 3.2–6.9 μm diam, binding hyphae thick-walled, strongly branched, non-septate, 4.0–6.3 μm diam.

*Cultures*: Colonies reaching 4–6 cm diam after 5 d at 32 °C on 2% MEA; mycelium at first white, the cream to orange pinkish buff, reverse cream to orange, with felty to cottony consistency and fluffy surface structure. Generative hyphae with clamp connections, skeletal and binding hyphae readily formed, diam. of skeletal hyphae 1.3–3.5 μm, thick-walled, wall with yellow-ochraceous pigment. Inflated intercalary and terminal elements readily formed at temperatures of 32 °C and higher.

*Habitat and distribution*: On trunks of *Quercus cerris, Q. pubescens, Castanea sativa, Carpinus betulus, Platanus acerifolia,* and *Populus* spp*.*, exceptionally also *Cerasium avium* and *Abies alba.* Based on sequences deposited in public databases, it occurs in Italy, Slovakia, Slovenia, Switzerland, United Kingdom, France, China and Iran**.** It is likely to be present through the whole Mediterranean area on suitable hosts, but is often misidentified as *F. fomentarius* (cfr, distribution of *F. fomentarius* shown in Bernicchia 2005).

**Fig. 10:**
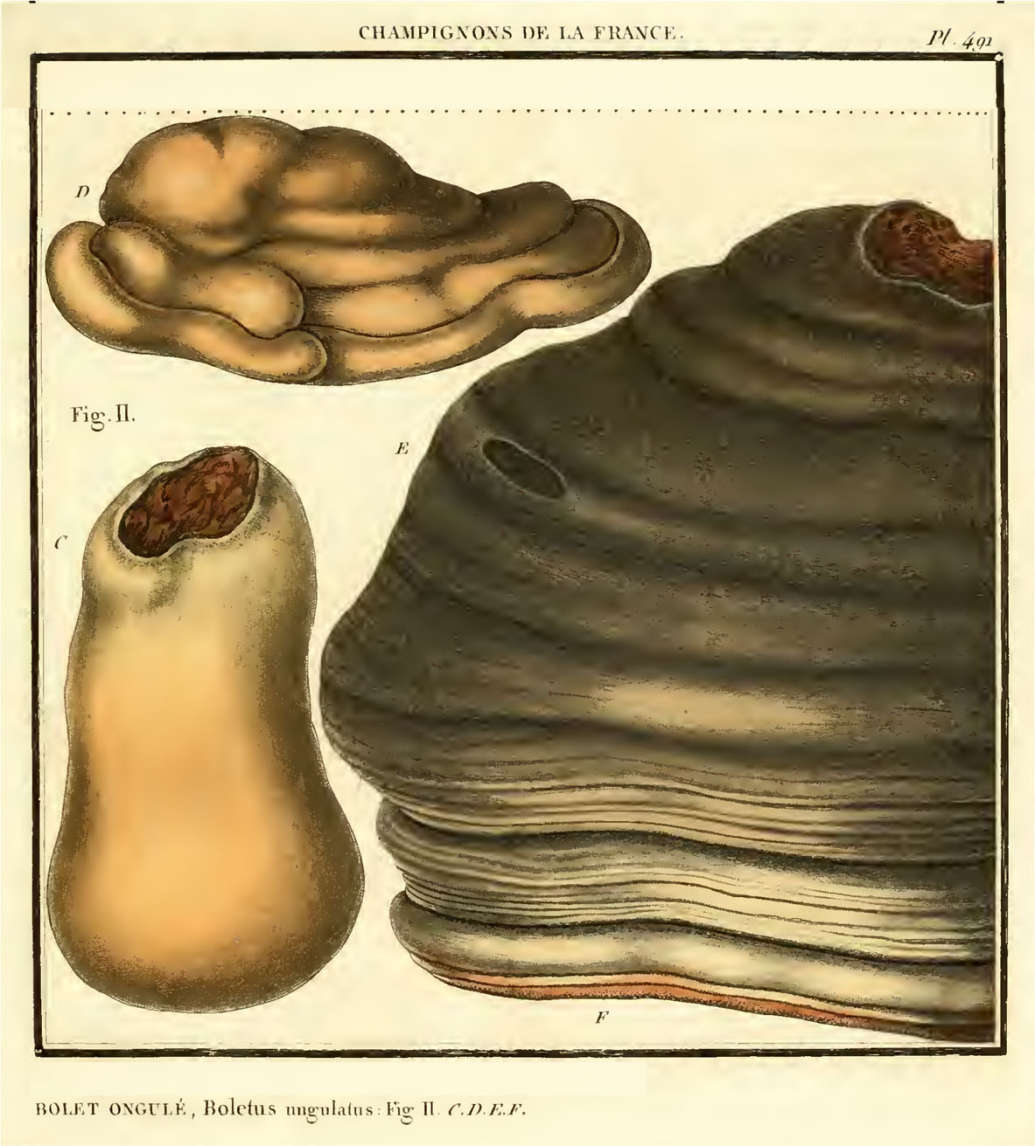
*Fomes inzengae*: basidiome slice of *Polyporus inzengae* no. 636 (lectotype) with hand-written label and printed protologue (cut out from *Erb. critt. Ital., ser. 1*). The lectotype is currently secondarily intermixed with another series “Mycotheca Universalis” (SIENA). Bar = 1 cm

*Nomenclature*: *Fomes inzengae* has long been regarded as a synonym or form of *F. fomentarius* (Bondartsev 1953; Domański et al. 1967; Donk 1933, 1974; Lécuru et al. 2019; Pilát 1941; Saccardo 1881). The basionym *Polyporus inzengae* is based on material collected and documented by Giuseppe Inzenga, who sent his material to De Notaris for identification. Cesati and De Notaris published the name with a printed description as no. 636 (see Fig. 10) in *Erbario Crittogamico Italiano* (Società crittogamologica italiana 1861; Sayre 1969), basing the description on the notes later reworked and twice published by Inzenga (1865, 1866) himself. Inzenga collected *P. inzengae* from *Populus dilatata* (now *P. nigra*) in Palermo (Italy, Sicily). The description from the protologue and description and illustrations from Inzenga’s *Funghi Siciliani* in black and white (Inzenga 1865: 17, pl. 2 Fig. 1) and reproduced in colour (Inzenga 1866: pl. 7 Fig. 1), agree with our concept of the Mediterranean *Fomes* lineage*.* Donk (1933) believed this was a milky white form of *F. fomentarius*, and others in the 20th century followed.

The original basidiome collected by Inzenga was cut into slices and sent to various herbaria as parts of an exsiccatae set. One part of this original collection no. 636 was later inserted in another set, the *Mycotheca Universalis*, conserved in Herbarium Universitatis Senensis (SIENA). This collection is interpreted as a syntype (cf. Wetzel and Williams 2018) and is here selected as the lectotype for the name; all other parts deposited elsewhere are therefore now isolectotypes. Cooke (1885b) transferred the name to *Fomes* in a list that was a continuation of *Fomes* species started in a previously published fascicle (Cooke 1885a) and is considered to have done so validly (Turland et al. 2018: Art. 35.1 Ex. 5).

The lectotype of *Fomes inzengae* is damaged by insects, but important diagnostic characters can still be evaluated: the hymenophore has 33–40 pores / cm, and the diameter of the skeletal hyphae ranges from (3.4–) 4.5–7.8 (− 10.0) μm (*n* = 30) with a mean value of 6.2 μm. A second collection of *F. inzengae* (*Erb. critt. Ital*. no. 977) collected in 1871 on *Quercus* (San Giuliano dal Sanno, Prov. Campobasso, Italy) has 32–38 pores / cm in the hymenium, and the skeletal hyphae range from 5.9 to 8.3 (− 9.4) μm. Unfortunately, we could not amplify DNA from these original collections of *Fomes inzengae,* and therefore we designate an epitype to fix the application of the name. Piccone (1876) recorded additional information on the second collection by Pedicino noting that it had also been included in Rabenhorst’s (1872) *Fungi Europaei exsiccati* no. 1508, which also consists of slices. Pedicino (1876) went on to record further observations.

*Comments*: *Fomes inzengae* has considerably smaller basidiospores than *F. fomentarius.* However, spores are difficult to observe in many pluriannual polypores because they are formed either in small quantities or during special, restricted seasonal periods. Additional characters, which are always present, are therefore crucial to distinguish these taxa: *Fomes inzengae* basidiomes can be separated from those of *F. fomentarius* on hymenophore pore size, and the diameter of skeletal hyphae. Moreover, substrate, growth rates, and volatile metabolites as well as pure culture characteristics help to distinguish these sister taxa. Barcoding rDNA ITS sequences are informative for species distinction in *Fomes*.

Additional specimens examined: **Italy**: *Prov. Siena*: Radicondoli, Riserva Naturale Cornocchia, on living tree of *Quercus cerris*, 29 Oct. 2013, *M. N. D’Aguanno* (IB20130333); *loc. cit.,* on *Q. cerris,* 26 Oct. 2016, *C. Perini, R. Kuhnert-Finkernagel & U. Peintner* (IB20160343); *loc. cit*., on living tree of *Q. cerris*, 1 Dec. 2017, *C. Perini* (IB20170300); Monticiano Riserva Naturale di Tocchi, on *Castanea sativa,* 28 Oct. 2016, *C. Perini, R. Kuhnert-Finkernagel & U. Peintner* (IB20160349); *loc. cit*., on dead deciduous tree, 28 Oct. 2016, *C. Perini, R. Kuhnert-Finkernagel & U. Peintner* (IB20160350); *loc. cit*., on *Carpinus betulus*, 28 Oct. 2016, *C. Perini, R. Kuhnert-Finkernagel & U. Peintner* (IB20160351); *loc. cit*., on *Quercus cerris*, 14 Jan. 2017, *C. Perini* (MSIENA8138); *loc. cit*., on living tree of *Quercus pubescens*, 14 Jan. 2017, *C. Perini* (MSIENA8062). *Prov. Campobasso*: San Giuliano dal Sanno, on *Quercus,* Sep.1871*, N. Pedicino* (SIENA, *Mycotheca Univ.*, *Erb. critt. Ital*. no. 977).

**Fomes fomentarius** (L.) Fr., *Summa veg. Scand*. **2**: 321 (1849); nom. sanct. *Syst. mycol*. **1**: 374 (1821)

*Basionym*: *Boletus fomentarius* L., *Sp. Pl.* **2**: 1176 (1753).

(Figs 4, 11)

**Fig. 11:**
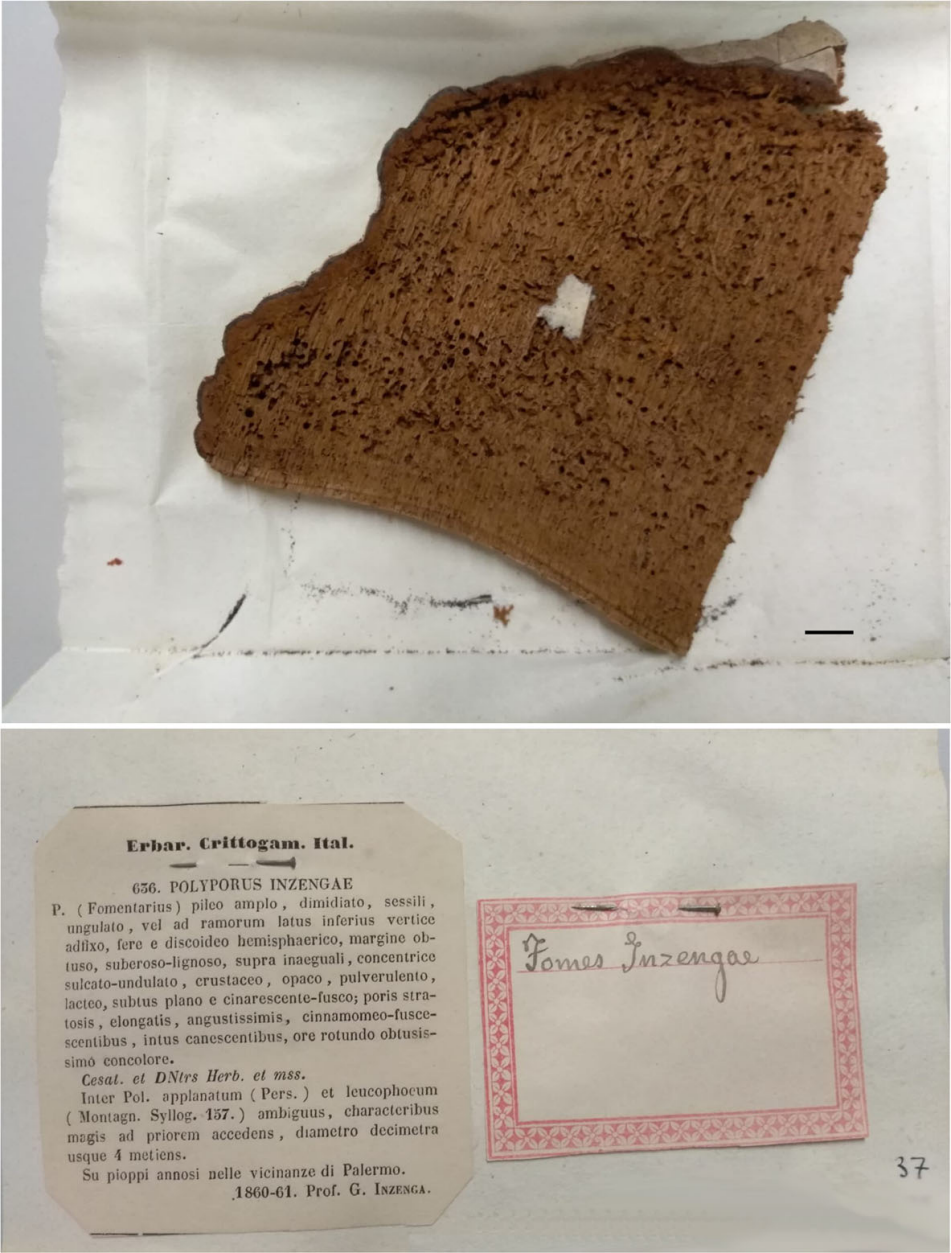
*Fomes fomentarius* **(**Bulliard tab. 491, fig. II C–F, 1791 – lectotype; as *Boletus ungulatus*). Parts of the original plate including another fungal species as well as the respective legend (orginally labeled Fig. I) were digitally removed. Reprint based on an original of Bulliard deposited in the New York Botanical Garden, The LuEsther T Mertz Library. Scanned version: 10.5962/bhl.title.5365

*Type*: Bulliard, *Herb. Fr*. tab. 491 fig. II C–F (1791, sub *Boletus ungulatus* Bull. (***lectotypus hic designatus*** IF556624) (Fig. 11). **Austria**: *Tirol*: Innsbruck, Magdeburger Hütte, alt. 1300 m, on living *Fagus sylvatica*, 20 Jul. 2013*, K. Rosam & U. Peintner,* (IB20130019, ***epitypus hic designatus,*** IF556623; GenBank KM360127 (ITS)).

*Diagnosis*: *Fomes fomentarius* basidiomes usually form on *Fagus* or *Betula* in boreal or temperate habitats. The pluriannual basidiomes have hymenophores with 27–30 pores / cm; the basidiospores are 12–18 × 4–7 μm.

*Description*: *Basidiomes* perennial, sessile, ungulate, tough, woody, to 25 cm wide. Upper surface quickly developing a glabrous greyish crust. Margin light brown, minutely tomentose; pore surface concave, pale brown, pores circular, 27–30 pores / cm, with thick tomentose dissepiments. Tube layers indistinctly stratified, reddish brown and becoming filled; context tissue a layer between the surface crust and the tubular layers, yellowish brown, tough, azonate. Granular core developing at the upper part of the context next to the substrate. *Basidiospores* cylindrical**,** hyaline, smooth, not amyloid, (12.5–) 13.5–18 (− 20.5) × 4.5–6.5 (− 7.5) μm, *Q* = (2.5–) 3.0–3.6 (− 3.5); *n* = 480. Usually produced in the spring in large quantities, difficult to observe during the rest of the year. Hyphal system trimitic, skeletal hyphae thick-walled, non-septate, with yellowish brown wall in 3% KOH, 3.0–6.4 μm diam, binding hyphae thick-walled, strongly branched.

*Pure cultures*: Colonies reaching 2–4 cm diam after 5 d at 32 °C, mycelium first white, the cream to orange-pinkish buff, reverse cream to orange, with a velutinous-felty to cottony consistency. Generative hyphae with clamp connections, skeletal and binding hyphae readily formed, skeletal hyphae 1.5–3.7 μm diam, thick-walled, wall with yellow-ochraceous pigment. Inflated intercalary and terminal elements formed at temperatures > 32 °C.

*Habitat and distribution*: In temperate habitats associated with *Fagus sylvatica*, and *Betula* spp., occasionally also with *Picea abies, Acer negundo, Populus* sp*.* or *Alnus incana.* Widely distributed in northern and central Europe, including Latvia and Russia. In Russia also on *Quercus*. The records from Russia and Alaska (*Betula neoalaskana*) indicate a potential circumpolar distribution. Occurring also in southern Europe on *Fagus*.

*Comments*: *Fomes fomentarius s. str*. is a temperate species with distinct morphological characters and host preference for *Fagus* and *Betula,* but in Russia it also grows on *Populus* and *Quercus*. The original diagnosis of Linné (1753) refers to a polypore growing on *Betula*. Fries (1821), in the sanctioning work, described the fungus as growing on *Fagus*. He also mentioned its use as tinder and as remedy against bleeding: “*pro fomite aptissima*. *In haemeragiis laudatus*”. He also cited several illustrations, which can be used to select a lectotype as under Art. F.3.9 material cited in the protologue of a sanctioning work is treated as original material for the purposes of lectotypification. The illustration published by Bulliard (1791) was selected as lectotype here as it best represents the current concept of *Fomes fomentarius*. Moreover, it is easily available online (10.5962/bhl.title.5365). A epitype is designated here in order to precisely fix the application of the name. We selected a collection from Austria on *Fagus* as epitype because all data are available for this collection, including a pure culture.

*Additional specimens examined*: **Austria**: Tirol, Achenkirch, Christlum, on *Fagus,* 26 Aug. 1991, *U. Peintner* (IB19910934); *loc. cit.,* on *Fagus,* 21 May 2017, *U. Peintner* (IB20170012); Gnadenwald, Gunggl, towards Maria Larch, on *Fagus*, 1 May 1991*, U. Peintner* (IB19910047); Innsbruck, Hötting, alt. 817 m, on *Fagus*, 10 Jul. 2013, *K. Rosam & U. Peintner* (IB20130011, IB20130016); *loc. cit.,* Stangensteig, alt. 820 m, on *Picea,* 25 Sep. 2013, *K. Rosam & U. Peintner* (IB20130022); Kärnten, Eberstein, on *Fagus sylvatica*, 13 Jun. 1990, *U. Peintner* (IB19901036). **– Finland**: Utsjoki, Kevo, Kevojokki, on dead *Betula,* 18 Aug. 1998, *M. Moser* (IB19980038). Sweden, Småland, Femsjö, Hägnan, *Fagus,* 21 Aug. 1976, M. Moser, IB19760143. **– Italy:** Corleto Monforte, Salerno, Parco Nazionale del Cilento e Vallo di Diano, 12 May 2008, *Pecoraro* (MSIENA8156); *loc. cit*., 12 May 2008, *Pecoraro* (MSIENA8157); *loc. cit*., 12 Nov. 2014, *M. N. D’Aguanno* (IB20140121). **– Russia**: *Moskow Oblast:* on *Betula,* 18 Oct. 2014, *A. Shiryaev* (SVER 926310); *Sverdlovsk Oblast,* Ekaterinburg City, on *Betula*, 4 Oct. 1978, *N.T. Stepanova-Kartavenko* (SVER 49614); *loc. cit., Populus*, 4 Aug. 1973, *A. Sirko* (SVER 10032); *Orenburg Oblast*, Orenburg State Nature Reserve, *Populus*, 1 Oct. 2017, *A.G. Shiryaev* (SVER 926313); *Volgograd Oblast*, Volzhsky, *Populus*, 8 Oct. 2001, *A.G. Shiryaev* (SVER 420865); *Novgorod Oblast*, Ilmen, *Populus*, 18 Aug. 1973*, N.T. Stepanova-Kartavenko* (SVER 229302); *Smolensk Oblast*, Dneper valley, *Populus,* 26 Sep. 2016, *A.G. Shiryaev* (SVER 867100); *loc. cit.,* Vyazma, *Quercus robur*, 22. Aug. 1978, *V. Ipolitov* (SVER 155532); *Samara Oblast*, Zhiguli Nature Park, *Q. robur*, 10 Sep. 1983, *F. Igorev* (SVER 303495); *Bashkiria*: on *Betula*, 18 Aug. 1963, *N.T. Stepanova-Kartavenko* (SVER 19051); *loc. cit.,* Nature Park Bashkiria, *Q. robur*, 19 Aug. 2012, *A.G. Shiryaev* (SVER 926313); *Krasnodar Krai*, on *Betula*, 5 Oct 1975, *N.T. Stepanova-Kartavenko* (SVER 22302); *Perm Krai*, Solikamsk, *Populus*, 23 Sep. 1999, *A.G. Shiryaev* (SVER 72466); *Kabardino-Balkar Republic*, *Q. robur*, 27 Sep. 2006, *A.G. Shiryaev* (SVER 784532); *Karelia Republic*, Kivach Nature Reserve, *Betula*, 20 Sep. 2017, *A.G. Shiryaev* (SVER 926311); *Tatarstan Repubic*, *Betula*, 30 Sep. 1971, *A. Sirko* (SVER 38225).

## DISCUSSION

### Cryptic species revisited

The rDNA ITS region has been accepted as the barcoding gene for fungi (Schoch et al. 2012), and molecular phylogenetic methods are now widely applied for distinction and definition of fungal taxa. This has led to the description of cryptic species representing distinct phylogenetic lineages (Krüger et al. 2004; Geml et al. 2006; Balasundaram et al. 2015; Obase et al. 2016; Sanchez-Garcia et al. 2016; Dowie et al. 2017; Mukhin et al. 2018). Meanwhile, multi-gene phylogenies have proven to be especially reliable for species definition, confirming several of these cryptic taxa, as in *Amanita* and *Fomes* (Pristas et al. 2013; Balasundaram et al. 2015). In this context it is especially important to screen for distinguishing characters, and to test them in a statistically significant number. This is tedious and time consuming, and thus not often carried out. In this study we focussed on cryptic species in the genus *Fomes*, in search of characters which allow an easy, fast and reliable distinction of these “cryptic” taxa without a need to sequence. We based our evaluation on classical characters in addition to several that have previously rarely been used for species delimitation. Our results show that cryptic species can be recognized in *Fomes* by micromorphological features, so providing valuable tools for a future more secure identifications of species in this important group of wood-degrading fungi.

### Basidiospores and hymenophoral pore size

When considering classical characters of basidiome morphology, basidiospore size and shape were clearly confirmed as valuable and important characters for the delimitation of species. However, basidiospore size can be an overlapping character in closely related species, or in species with a wide basidiospore size ranges. *Fomes inzengae* basidiospores are significantly smaller (9–12.5 × 3–4 μm) than those of *F. fomentarius.* The latter have been reported to have a very wide range, e.g. 16–24 × 5.5–6.5 (Jülich 1984), 18.5–19 × 5.5–6.0 μm (Breitenbach & Kränzlin 1986), 12–18 (20) × 4.0–7.0 μm (Ryvarden & Gilbertson 1993, 1994), or 12–15 (18) × 4.5–7.0 (Bernicchia 2005). *Fomes fasciatus* basidiospores are reported as 12–14 × 4.0–4.5 μm (Gilbertson & Ryvarden 1986). Even for large spores, the distinction of *F. inzengae* is always possible on spore width alone.

Polypore basidiomes often do not form basidiospores throughout the year, making it difficult to use them. As in many other polypores, *Fomes* basidiospores can be detected only during short periods, such as spring, or similar periods without water or temperature stress. It is therefore important to find additional characters that can be used throughout the year. Hymenophore pore diameter emerges as such an important and reliable morphological character for the delimitation of taxa in *Fomes*. However, data need to be measured in a statistically relevant numbers, and under a stereomicroscope. Hymenophore pore diameter is not necessarily an independent character: we first hypothesized that hymenophore pore size could be positively correlated to basidiospore size. *Fomes inzengae* has smaller basidiospores and also smaller hymenophoral pores then *F. fomentarius*. However, *F. fasciatus* has even smaller pores (4–5 / mm), although having intermediately sized spores. This type of correlation would be worthwhile to test in a wider range of polypore genera. Basidiospore size has been related to the size of the basidiomes and to the life-style of different polypore genera (Kauserud et al. 2008, 2011).

### Skeletal hyphal diameter

The diameter of skeletal hyphae also turned out to be a valuable character for the delimitation of species in *Fomes* when measured in a statistically significant number. In naturally grown basidiomes, *F. inzengae* has significantly thicker skeletal hyphae than *F. fomentarius*. The diameter of skeletal hyphae is generally significantly smaller when measured in pure culture, reaching only about half that of skeletal hyphae in basidiomes. Moreover, our pure culture experiment confirms that morphological characters are dependent on environmental characters such as temperature. Also, in pure culture, skeletal hyphal diameter is still significantly different between the two *Fomes* species, but it is reversed. In pure culture, *F. fomentarius* always has significantly thicker skeletal hyphae than *F. inzengae.*

The morphology of fungal pure cultures from wood-inhabiting fungi was described for more than 1000 isolates (Stalpers 1978), but a comparison to structures in the basidiome was not carried out. Cultivation was carried out on MEA 2% and isolates were incubated at room temperature and daylight. The culture diameter of *F. fomentarius* was reported to be 40– > 70 mm after 7 d. These data cannot easily be compared due to differences in incubation times; because of the fast growth of *F. inzengae,* we measured culture diameter after 5 d*.* The reported diameter of the skeletal hyphae (1.5–3 (− 4) μm) is within the range of our data, but a distinction is not possible due to lack of statistically relevant data. The inflated intercalarly and terminal elements, as observed in our pure cultures, were also reported by Stalpers (1978); he called them “cuticular cells”.

A comparison of skeletal hyphal diameter reported for pure cultures (Stalpers 1978) and basidiomes (Gilbertson & Ryvarden, 1986) confirms that skeletal hyphae of polypores are usually thinner in pure cultures than in the basidiomes (e.g. *Fomitopsis pinicola* 1.5–2.0 vs. 3–6 μm, *Gloeophylum abietinum* 2–4 vs. 3–6 μm, *Lenzites betulina* 1–4 vs. 3–7 μm, *Trametes gibbosa* 1.5–3.5 vs. 4–9 μm). Skeletal hyphae have an important structural function in basidiomes: thicker skeletal hyphae provide more stability and durability. Moreover, time could also be an important factor influencing the diameter of structural hyphae.

### Growth characteristics in pure culture

Growth characteristics in pure culture, growth rates, and optimum growth temperatures are important characters for the delimitation of species in polypores (McCormick et al. 2013; Dresch et al. 2015). However, methods need to be standardized in order to obtain a meaningful comparison of results. We propose using daily growth rates as a meaningful and easy measure for colony growth under standardized conditions. *Fomes inzengae* has an optimum growth temperature of 30 °C, with growth rates of 1.46 ± 0.20 cm / d. *Fomes fomentarius* has an optimum growth temperature of 25–30 °C, with significantly slower growth rates of 1.11 ± 0.80 cm / d at 30 °C. It is difficult to compare our growth rate data with that from other studies, but the optimum temperature is clearly higher for *F. fasciatus*, ranging between 32 and 39 °C (McCormick et al. 2013).

### Volatile organic compounds

Fungi emit a large spectrum of volatile organic compounds (VOCs). Recent studies have shown that fungal emission patterns can be species-specific, and chemotyping is possible for some species and functional groups (Müller et al. 2013; Redeker et al. 2018). Species-specific VOCs have already been defined for a few polypore species (Marshall 1970; Cowan 1973; McAfee & Taylor 1999; Rapior et al. 2000; Rosecke et al. 2000; Ziegenbein et al. 2010; Konuma et al. 2015). More generally, this confirms that direct mass spectrometry allows for a reliable species identification of wood decaying polypores, including a discrimination between *F. fomentarius* and *Fomes inzengae* (Pristas et al. 2017).

Differences in the production of VOCs observed between fungal basidiomes and pure culture are striking. At first, it is surprising that pure cultures produce a higher diversity and higher concentrations of VOCs than basidiomes. Wood-decaying fungi produce specific VOCs during wood degradation, and emission patterns depend on both the cultivation stage and the substrate (wood chips or potato dextrose agar), suggesting that wood degradation might activate synthetic pathways such as VOC production (Konuma et al. 2015). Emission patterns of basidiomes could differ because hyphae are not physiologically active any more: no wood degradation occurs in basidiomes, and in those the hyphae have mainly structural (skeletal hyphae) and reproductive functions. Thus, functional traits are different in basidiomes, and they can be detected by VOC emission patterns. Moreover, VOCs have also been proposed as important substances for the interaction with other organisms (Chiron & Michelot 2005; Morath et al. 2012; Bennett & Inamdar 2015; Elvira Sanchez-Fernandez et al. 2016), and interactions in the substrate are clearly different from those in basidiomes.

### Substrate utilization

Our data confirm host substrate as important driver of speciation in wood degrading polypores (Kauserud et al. 2007; Skaven Seierstad et al. 2013). Long distance spore dispersal appears to be common in wood-degrading fungi (Moncalvo & Buchanan 2008; James 2015), explaining the Northern Hemisphere distribution of the genus *Fomes*. However, basidiospores can only establish on a suitable substrate, as shown by our data: we collected and isolated typical *Fomes fomentarius* on *Fagus* growing in southern Italy. Especially in white-rot lineages, host switching often leads to specialization to an angiosperm substrate, and thus to speciation (Krah et al. 2018). Substrate utilization reflects enzymatic capacities and the fungal metabolic properties. Host switches occur only rarely, and if no suitable host is available. Based on the available distributional data, it can be assumed that the ability to degrade different wood types is an important driver for speciation in *Fomes*.

### Functional implications of the differences between *F. inzengae* and *F. fomentarius*

The differences detected between the two species of *Fomes* reflect an optimal adaptation to environmental conditions. *Fomes inzengae* appears to be well adapted to a warm and dry climate, and to the degradation of difficult substrates containing a wide array of antifungal substances, such as oak wood. The optimum growth temperature is higher, and ground basidiomes impressively show the ability of the tissues to absorbs water like a sponge. We speculate that the larger diameter of skeletal hyphae and a less hydrophobic surface of hyphae might be responsible for this particular property. *Fomes inzengae* is richer in VOCs, indicating a highly active and versatile natural product profile.

### Potential diversity in the genus *Fomes*

The genus *Fomes* was originally circumscribed by Fries (1849, 1874) in a much wider sense than today, but the actual concept of the genus *Fomes s. str.* includes a comparatively low species diversity (Justo et al. 2017) (Lowe 1955; Gilbertson & Ryvarden 1986, 1987; Ryvarden & Gilbertson 1993, 1994; McCormick et al. 2013).

*Fomes graveolens* (syn.: *Globulifomes graveolens*) is as potential sister taxon of *F. inzengae* based on analysis of a short ITS sequence (MG663229), but more data are needed for an exact placement and delimitation of this species.

*Fomes fasciatus* can easily be delimited based on the applanate-dimidate basidiomes and in growing on subtropical hardwoods in the southeastern USA. Delimitation can also be based on pore diameter, basidiospores size, and the optimum growth temperature of isolates: *Fomes fasciatus* basidiomes have (3–) 4–5 pores / mm, the basidiospores are in the range 7.50–16.25 × 2.50–6.25 μm, mean 10.85 ± 0.10 × 4.15 ± 0.70 μm (*n* = 230), and the optimum growth temperature for isolates is higher than 30 °C.

However, our and other previous phylogenetic analyses indicate that *Fomes* diversity is higher than currently assumed (McCormick et al. 2013; Pristas et al. 2013). Phylogenetic analyses indicate at least one new *Fomes* species from Asia, and a potential new species from North America (*F. fomentarius* II in McCormick et al. 2013). Hymenophores of *F. fomentarius* II from North America have 2–4 (− 5) pores / mm, and basidiospores in the range of 10.0–21.3 × 2.5–7.5 μm, mean 17.55 ± 0.05 × 5.27 ± 0.03 μm (*n* = 805). Delimiting characters such as pores / cm and spore size overlap between the two lineages of *F. fomentarius*, and further comparative analyses (e.g. VOC profiles of basidiomes or culture, or the diameter of skeletal hyphae) are needed to clarify whether *F. fomentarius* II is a distinct species or not. Finally, a BLAST analyses of ITS sequence (HM136871), the *Fomes* species reported from Mexico, reveals that collection does not belong to the genus.

### Available epithets for *Fomes* lineages

*Fomes fomentarius s. lat.* Has a large number of synonyms, some of which could provide epithets for naming new *Fomes* lineages. For example, *F. excavatus* (syn. *Polyporus fomentarius* var. *excavatus*) described on birch from Isle a la Crosse in Saskatchewan, Canada, and might possibly represent the North American clade of *Fomes* or some other genus. The original description (Berkeley, 1839) corresponds to *F. fomentarius s. lat*. However, the information provided, “Pores small, perfectly round, fawn-coloured, cinnamon within.”, does not permit a distinction of *Fomes* taxa. Original material needs to be studied in order to test whether the distinguishing characters for basidiomes defined in this study (e.g. pore size, skeletal hyphae diameter, spore size or production of VOCs) enable an unambiguous characterization of this North American *Fomes* taxon to be made.

## Conclusions

Based on the proposed morphological and physiological characters, it should be easily possible to delimit new lineages of polypores as valid, and distinct species, in order to minimize the number of cryptic lineages in polypores. We also point out, that it is important to consider epithets, which were previously synonymised, as potentially available names for newly recognized phylogenetic linages. Several morphological characters have been shown to be important and taxonomically valuable if evaluated in statistically relevant numbers, e.g. hymenophore pore diameter or diameter of skeletal hyphae. Physiological characters turned also out to be species-specific in this case, notably the daily mycelial growth rates, or temperature range of pure cultures. The production of volatile organic compounds also emerges as a promising tool for fast and reliable species delimitation in the future.

## Data Availability

All data generated or analysed during this study are included in this published article [and its supplementary information files].

## Abbreviations

BPP: Bayesian Posterior Probabilitiy
ESS: Estimated Sample Size
F: *Fomes*
MEA: Malt extract agar
MCMC: Markov Chain Monte Carlo
ML: Maximum Likelihood
PSRF: Potential Scale Reduction Factor
PCA: Principal Component Analysis
p: Probability value
PTR-TOF-MS: Proton Transfer Reaction Time of Flight Mass Spectrometer
rDNA ITS: Ribosomal DNA internal transcribed spacers
s. l.: Sensu *lato*
SPR: Subtree-Pruning-Regrafting
VOC: Volatile organic compound
w/v: Weight to volume ratio
w/w: Weight to weight ratio
